# Certified Medical Interpreters’ Perspectives on Relationship-Centered Communication in Safety-Net Care

**DOI:** 10.15694/mep.2018.0000169.2

**Published:** 2018-10-24

**Authors:** Neda Ratanawongsa, Angelica Cardenas, Bruce Occeña, Jeff Critchfield, Mary Mercer, Kara Myers

**Affiliations:** 1 University of California; 2 University of California; 3 Zuckerberg San Francisco General Hospital and Trauma Center; 4 San Francisco Department of Public Health

**Keywords:** Communication Barriers, Cross-Cultural Care, Physician-Patient Relations, Professional-Patient Relation, Teamwork, Interdisciplinary Communication, Safety-net Providers

## Abstract

This article was migrated. The article was marked as recommended.

**Background:** Interpreters may offer valuable perspectives on ways clinicians could improve communication skills. Relationship-centered communication (RCC) curricula aim to promote effective communication between patients and clinicians and among members of health care teams.

**Methods:** We conducted a 90-minute workshop with certified interpreters at an academically affiliated safety-net system to solicit feedback on content offered during RCC skills trainings. We applied an editing analysis style to transcribed quotes to reveal opportunities to optimize RCC skills trainings for application in interpreted interactions to improve safety-net care for diverse populations.

**Results:** Twenty-two Spanish-, Cantonese-, Mandarin-, Vietnamese-, and Russian-speaking interpreters participated. Overall, interpreters emphasized the importance of creating a supportive environment for safety-net patients. One Spanish-speaking interpreter added: “When they get up in the morning and go to work, they may get deported. So, that’s important to create an atmosphere to help them open up. And they may tell you stuff that’s directly pertinent to patient care.” Thematic analysis revealed opportunities to tailor and reinforce each RCC stage. On agenda-setting and rapport-building: “We need a little background on the phone, and we don’t know how many people are in the room ... Sometimes you’re talking to the mom, but the doctor didn’t even bother to say it.. [If] we’re lost, we’re bound to make mistakes.” On eliciting the patient’s perspective: “Start with this information so they know you’re still going to give them your advice: “I’m going to let you know what I think is going on, but what do you think is going on?” On negotiating a shared plan: “[Teachback] is really important. Otherwise it puts an incredible burden on the interpreter ... I’m not sure that the patient really understood.”

**Conclusions:** Teaching RCC in partnership with medical interpreters could provide opportunities to deepen clinician RCC skills for more effective patient-interpreter-clinician interactions.

## Introduction

Effective clinical communication is crucial for patients with limited English proficiency (LEP), who experience higher risks for poorer healthcare and health outcomes ( Parker et al 2017, Kim et al 2017). In the United States, certified medical interpreters are vital care team members in safety-net health systems, which are public health care systems that rely heavily on governmental funding, through programs like Medicaid and Medicare, to provide care to socioeconomically disadvantaged populations ( Chen and Jacobs 2016). Interpreters’ involvement in care has been associated with improved patient knowledge, patient satisfaction, and quality of care ( Chen and Jacobs 2016, Flores 2015). Medical interpreters may experience dilemmas during suboptimal patient-clinician interactions, and interpreters can offer unique insights into challenges and opportunities for improving communication for LEP populations ( Hsieh 2006, Hsieh 2008, Hsieh 2010).

Relationship-centered care is a framework for effective communication across all participants, including patients and health care team members ( Beach and Inui 2006). Relationship-centered care is founded upon principles that acknowledge the importance of affect and emotion in these relationships and the influence each person has on one another ( Beach and Inui 2006). This framework differs from patient-centered care in the “explicit recognition that clinicians bring their personhood into the encounter,” emphasizing the importance of the clinicians’ emotions, authenticity, and self-care not only in their relationships with patients but also with other care team members ( Beach and Inui 2006). Health care systems across the United States have implemented relationship-centered communication (RCC) trainings, using methods combining interactive didactics, demonstrations, skills practice using stories from personal experiences, and facilitated small group feedback, and such systemic implementation has been associated with improved patient and provider satisfaction ( Chou and Cooley 2017, Boissy et al 2016).

Given the importance of both interpreter-facilitated and relationship-centered communication to safety-net care, we conducted a workshop to elicit interpreters’ feedback about optimizing RCC skills trainings for application in interpreted interactions to improve safety-net care for diverse populations.

## Methods

We conducted a thematic analysis of perspectives elicited during a 90-minute interactive workshop to solicit feedback on RCC training content from certified medical interpreters at an academically affiliated, safety-net system. These interpreters deliver in-person, telephonic, and video medical interpretation services across all inpatient and outpatient settings in a city with 45% non-English speaking households. The workshop occurred in October 2017 during the Interpreter Services’ monthly professional development series. RCC trainings for clinicians, clinical and operational staff, and intact clinical teams began in 2013. Workshop facilitators had standardized training in teaching RCC.

The workshop began with a reflection exercise in which interpreters broke into pairs to share a past experience in which the patient-clinician-interpreter communication was effective. After debriefing these stories for unifying themes, the facilitators presented three mini-didactics and demonstrations of relationship-centered communication skills at each stage of a clinical interaction: 1) Agenda-setting and rapport-building (building rapport quickly, eliciting all of the patient’s agenda items, negotiate the agenda, and introducing the computer); 2) Eliciting and responding to the patient’s perspective (exploring the patient’s function, ideas, fears, and expectations and demonstrating empathy verbally and non-verbally); and 3) Negotiating a shared plan (sharing information, assessing understanding, summarizing and clarifying, and closing the encounter) ( Chou and Cooley 2017). After each of the three demonstrations, the facilitators elicited feedback from the interpreters about what they found surprising or notable during the demonstrations and any challenges, concerns, or opportunities for using those skills in an interpreted patient-clinician interaction.

One researcher (NR) transcribed notes and quotes from participants in real-time during the workshop. Two researchers (NR, AC) used an editing analysis style to identify “meaningful units or segments of text that both stand on their own and relate to the purpose of the study” ( Strauss and Corbin 1998). In this data, individual quotes could represent more than one concept and be categorized by researchers under multiple different codes. We came to consensus in codes and themes and then selected representative quotes.

The UCSF Committee on Human Research categorizes this analysis as quality improvement work that did not require IRB approval or participant consent.

## Results

Of 37 certified medical interpreters, 22 attended the workshop: 11 for Spanish; 10 for Cantonese, Mandarin, and/or Vietnamese; and 1 for Russian. The other 15 interpreters were providing interpretation services or not working that day. None had previously attended any RCC trainings.

Prior to the focused discussion on RCC, interpreters shared stories in pairs and then debriefed with the larger group about what they have found to be the most effective communication involving patients, clinicians, and interpreters. Themes across these stories included clear, empathic communication and involvement of interpreters in the process of knowing patients as individuals with complex psychosocial needs. For example, one Cantonese-speaking interpreter said: “Our oncology care teams are very in tune with needs of patients, their wants, their doubts, they’re really keen; it doesn’t feel rushed; it doesn’t feel let’s get it done, let’s get over the science ... [They] take the time to send that message of empathy saying, ‘This team is doing whatever humanly possible to be there.’ I feel that’s very important, especially for our population that hasn’t had that contact ... It’s very impressive.” One Spanish-speaking interpreter added: “When they get up in the morning and go to work, they may get deported. So, that’s important to create an atmosphere to help them open up. And they may tell you stuff that’s directly pertinent to patient care.”

Table 1 describes categories and quotes for how each stage of the medical encounter might be tailored or emphasized, from the cultural and linguistic perspectives of the interpreters.

**Table 1.** Feedback elicited from certified medical interpreters about relationship-centered communication skills training for clinicians at an urban, academically-affiliated safety-net system

Section of the Medical Encounter	Tips	Quotes
**Agenda-Setting and Rapport-Building**	Introduce everyone in the room to the interpreter, including details that may be culturally and linguistically relevant.	Realize your interpreter has no eyes - you have to be their eyes and give enough information to understand the situation. Some providers are really nice, and the provider tells me who else is in the room. And we don’t say “You or me.” So, we need to know who is in the room and how old they are. For example, when I interpret for the patient, and the patient doesn’t answer, but someone else answers ...
	Take time to include interpreter in rapport-building.	They don’t even let the interpreter say hi and they’re giving the (medical record number) ...
	Be mindful of body language.	I think the palliative care team really stands out. Kindness. One provider who is always looking at the patient. If the patient is lower, the provider bends down to get to their level. Body language really makes a difference.
	Be transparent about time while eliciting the patient’s full agenda.	And I really liked letting people know that the time is only 15 minutes, sometime the provider finishes, and the patient comes up with another request, and another request. One time a patient had 18 requests, and the doctor addressed it, and it took more than an hour. I’ve seen this specific strategy of scaffolding recently a few times, and it really does make a difference. And I’ve seen a few patients list 5 things, but I’ve seen providers say, “Thing number 7 is really important, more important than the things from your last provider, so we’re going to start with that.” ... But in setting up the expectation that there are people after you.
	Be transparent about the “others” in the room, including the computer and supervisors / team members.	Patients really pick up when someone cares, as opposed to when someone may be just behind the computer. Some providers think, “Well the interpreter is going to say what I’m saying,” but it’s also important what they communicate. An explanation, transparency, “I’m going to chart some things now.” When he was doing the typing on the computer, he explained that. Many times, the doctors just do it. Instead of explaining what he’s doing and the role it plays. If they’re explaining something to a supervisor, please say that to a patient. Sometimes when we’re on the phone, we have to explain to the patient. But if they could just say, “Sir, I’m going to explain this to my supervisor.” Because otherwise they are thinking, “What’s going on here?”
**Eliciting and Responding to the Patient’s Perspective**	Use active listening.	The nurse just listened. Sometimes you can just put away the paper with the boxes and just get that information. She took her time and she listened and she would ask the next question ... And she would just ask something and just pause. I could only listen to the verbal. Wanting to know and listening.
	Ask about concerns / fears related to social determinants.	There was a young woman from Guatemala, and the nurse was doing an intake ... And we found out the woman walked all the way here, with her baby, and the nurse said, “What do you have on your ankle?” And we learned that she had been detained ...
	While eliciting patients’ ideas, remember some patients may need reassurance you will share your medical recommendations.	He took the liberty to ask the patient what they thought was going on. Giving them the power to say, “I think I know what’s going on.” And when a condition is happening, how bad it is affecting your routine, your lifestyle. That really adds a dimension to what’s going on. I like how he said, “You know your body the best.”
	Empathy statements can be very powerful.	I think in asking that patient how that affected their life, if there’s not an immediate solution, then you know they are thinking about another thing to alleviate their life. As a patient, I would feel grateful. He said, “I’m going to partner.” That’s really humble. In Chinese culture, that’s important. He stepped down his role. You can feel this doctor has a heart. We’re walking together. That’s a kind of trust-building.
**Negotiating a Shared Plan**	Assess and build a patient’s understanding of the reason to consider a treatment plan	The other thing is, every point when a decision was made, he didn’t just announce a decision. He gave some information about how he came to the decision. And that’s really important. The more a patient can understand about the medical process, the more comfortable they feel. It’s great, because you addressed it in the beginning, but you wrapped it up fine. Some doctors say, “Septra, 3 days, you’re done.” I’ve seen others say, “You have a UTI. Do you know what that is?” Because some patients know.
	Explain a treatment plan in a systematic, step-by-step, format.	Asthma clinic is really good at breaking it down. Sometimes it’s the nuances where things go wrong. How to use the spacer. I’ve noticed with the chemotherapy sessions. They take the time, they are really structured. Try to turn over every stone. The most important thing is that the patient understands, that it’s clear, that’s the message is brought home. We’ve also noticed, since this is a learning center, when someone is more veteran or attending vs. a resident or med student or someone coming through rotation. A lot of the understanding is lost because of the rush. No time to break down the medical jargon, and if you don’t get it, that’s too bad.
	Use visuals to help overcome communication barriers.	I was in intensive care ... they explained over and over again, and they didn’t get it. Those questions have been answered. Finally, you saw someone showing the picture, moved the bed for the patient to see the computer, “See the fluid” ... that’s why the spinal cord is injured, because of the cancer, and the compression.” Sometimes no matter how long you may not get it. How could they get sepsis? But if you see the video, maybe you can get it.
	Tailor the plan to a patient’s lifestyle and motivations.	And the part about asking about what they will drink ... Because those details are just as important as the pills you’re going to take. I hope that the doctor asks patients which pharmacy you want to send the medication to. They want to go to their own pharmacy in their own neighborhood where they don’t have to wait a long time. I also liked how he mentioned the family again. It was going back to the whole health, the health of the whole person. Going back to the family ... This may be a good time to spend time with your family.
	Teachback helps interpreters who feel concerned that patients don’t understand the plan.	I think it’s great. I never heard doctors do what you do. The last part when you asked, “I want to make sure you understand.” That was the best part. Take the time to make sure they know how to read and write. Some encounters say, “You just read the labels and you’re good to go.” And I have to make the leap of faith, and I find out they can’t read or write.

Organized by three stages of a relationship-centered encounter, reflections and suggestions included:

Agenda-Setting and Rapport-Building:

• Introduce everyone in the room to the interpreter, including details that may be culturally and linguistically relevant.
• Take time to include the interpreter in rapport-building.
• Be mindful of body language.
• Be transparent about time while eliciting the patient’s full agenda.
• Be transparent about the “others” in the room, including the computer and supervisors / team members.

One Cantonese-speaking interpreter said, “In Vietnamese and Chinese cultures, we don’t say, “you and me” we address by gender. If you are older, we say “Auntie” and if you’re younger, we say “sister.” So, we need to know the age of the patient.” Another interpreter agreed, “We need a little background when we are on the phone, and we don’t know how many people are in the room and who are they and how old are they. Sometimes you’re talking to the mom, but the doctor didn’t even bother to say it, but at least say, ‘We’re in the dermatology clinic, and we’re in the room with the mother and the son is bilingual.’ And we’re in the middle of it, and we’re just thrown in there ... We’re lost, and we’re bound to make mistakes.”

Eliciting and Responding to the Patient’s Perspective:

• Use active listening.
• Ask about concerns / fears related to social determinants.
• While elicit patients’ ideas, remember some patients may need reassurance you will share your medical recommendations.
• Empathy statements can be very powerful.

On the third point, one Russian-speaking interpreter shared, “It’s probably cultural ... when you ask a Russian-speaking patient, they say, “How would I know? I’m not a doctor?” Others nodded and murmured ascent (“They get upset!”). A Spanish-speaking interpreter suggested, “Start with this information so they know you’re still going to give them your advice. ‘I’m going to let you know what I think is going on, but what do you think is going on?”

Negotiating a Shared Plan:

• Assess and build a patient’s understanding of the reason to consider a treatment plan.
• Explain a treatment plan in a systematic, step-by-step, format.
• Use visuals to help overcome communication barriers.
• Tailor the plan to a patient’s lifestyle and motivations.
• Teachback helps interpreters who feel concerned that patients don’t understand the plan.

On the last point, one Spanish-speaking interpreter stated, “I think it’s really important, the teachback, because otherwise it puts an incredible burden on the interpreter, because otherwise it forces the interpreter into the role of a cultural broker, I’m not sure that the patient really understood ...”

## Discussion

Our findings reinforce the importance of including interpreters in relationship-centered care, emphasizing a parallel process for clinical care teams to employ RCC skills with the interpreters during the encounter. Interpreters validated the RCC framework for promoting high quality patient-clinician communication in general, but then offered specific context and tailoring of the different communication techniques throughout each RCC stage for interpreted interactions. Interpreters appreciated when clinicians went beyond logistics about a patient’s name and medical record number to set the stage for their interpretation, building rapport and orienting the interpreters to everyone in the room. In addition, interpreters felt intense empathy for the patient’s situation and appreciated the compassion they felt was conveyed by care team members. Finally, interpreters expressed a discomfort when they felt treatment plans were too complex for patients to understand or complete, and they felt relief when clinicians assessed patient understanding.

Interpreter services have positive impacts on the patient-centeredness of care, improving patient knowledge and satisfaction, clinician satisfaction, and process outcomes for quality of care ( Chen and Jacobs 2016, Flores 2005). However, interpreters experience challenges in the many roles they may play in the medical encounter. Hsieh described how interpreters see themselves in four types of roles: “conduit,” “advocate,” “manager,” and “professional” ( Hsieh 2008). So, interpreters do not see themselves as solely conveying information, but also ensuring the complete transfer of information (including non-verbal messages), managing the optimal exchange of the information, reinforcing the provider-patient relationship, and empowering patients ( Hsieh 2008). Thus, when training clinicians and care teams in the fundamental skills for RCC, educators need to acknowledge and reaffirm the importance of interpreters and partner with them to enhance RCC skills in this parallel process.

These findings build on best practice recommendations for clinical teams working with interpreters, such as maintaining eye contact and focusing communication directly with the patient, limiting the amount and complexity of information, attending to nonverbal cues, and using teachback to assess understanding ( Chen and Jacobs 2016). Meanwhile, the principles and teaching methodologies of RCC communications skills trainings - experiential learning using shared stories, roleplay practice, and facilitated small group feedback ( Chou and Cooley 2017, Boissy et al 2016) - offer a framework for interprofessional training in which interpreters and clinicians could offer one another feedback and share strategies over challenging patient-interpreter-clinician interactions.

### Limitations

The sample is small, and we were unable to capture the participating interpreters’ sociodemographic characteristics. Although the facilitators have expertise in soliciting diverse perspectives, some interpreters may not have shared perspectives, including ones that differed from others. Finally, as a single institution study, we cannot generalize our findings, but our sample was enriched through inclusion of interpreters from multiple languages and racial/ethnic backgrounds.

### Conclusion

Certified medical interpreters shared numerous strategies for tailoring the content and reinforcing the value of an RCC skills training for clinicians at an urban, academically-affiliated safety-net health system.Developing curricula for applying RCC in partnership with medical interpreters could provide opportunities to reinforce both RCC and interpreter-mediated communication skills, while strengthening the relationships across teams caring for diverse populations.

## Take Home Messages

• Interpreters, as vital team members caring for patients with limited English proficiency (LEP), offer valuable input for how clinicians can improve communications skills.
• Relationship-centered communication (RCC) skills trainings offer opportunities to improve skills with patient-clinician communication and to build relationships with team members like interpreters.
• In all stages of an encounter - agenda setting and rapport-building, eliciting and responding to the patient’s perspective, and negotiating a shared plan - interpreters offered advice for linguistic and cultural tailoring of RCC clinician communication strategies.
• These findings highlight how interpreters could be included in the development and implementation of RCC skills trainings in health systems serving LEP populations and the value of their inclusion.

## Notes On Contributors

Neda Ratanawongsa, MD, MPH, is an Associate Professor in the Division of General Internal Medicine, Department of Medicine, University of California, San Francisco (UCSF) and the UCSF Center for Vulnerable Populations at Zuckerberg San Francisco General Hospital and Trauma Center.

Angelica Cardenas, MPH is the Educational Coordinator and AHA Training Center Coordinator for the Department of Education and Training, Zuckerberg San Francisco General Hospital and Trauma Center, San Francisco Health Network, San Francisco Department of Public Health.

Bruce Occeña is the Director of Tele-health and Interpreter Services at the San Francisco Health Network, San Francisco Department of Public Health.

Jeff Critchfield, MD is the Chief Medical Experience Officer and Medical Director of Risk Management at the Zuckerberg San Francisco General Hospital and Trauma Center, and a Professor in the UCSF Department of Medicine.

Mary Mercer, MD, MPH is an Associate Professor and Director for Performance Improvement at the UCSF Department of Emergency Medicine, Zuckerberg San Francisco General Hospital and Trauma Center.

Kara Myers, CNM, MS is a professor in the Department of Obstetrics, Gynecology and Reproductive Sciences, Zuckerberg San Francisco General Hospital and Trauma Center.
