# Supplementary material for: Certified Medical Interpreters’ Perspectives on Relationship-Centered Communication in Safety-Net Care
Source: MedEdPublish (2016). 2018 Oct 24;7:169. Originally published 2018 Aug 14. [Version 2] doi: 10.15694/mep.2018.0000169.2 (PMC10701798; doi:10.15694/mep.2018.0000169.2)
Supplement: Peer review history

# Reviewer report: Reviewer response for version 2

**DOI:** 10.21956/mep.20230.r31342

**Published:** 2019-06-04

**Authors:** Ken Masters

**Affiliations:** 1 Sultan Qaboos University

This review has been migrated. The reviewer awarded 5 stars out of 5 This is the revised version of the paper, taking into account the previous reviewers’ comments. The authors have addressed the reviewers’ concerns, and have produced a much-improved paper. The paper shows the many complexities involved in working with interpreter services, especially the complexities in the relationship between language and cultural norms and practices, and the need for extremely careful and specialised training.This paper would be of importance for any medical staff working with interpreters, even in completely different cultural and language contexts.

Reviewer Expertise:

NA

No decision status is available

# Reviewer report: Reviewer response for version 2

**DOI:** 10.21956/mep.20230.r31343

**Published:** 2018-11-11

**Authors:** Trevor Gibbs

**Affiliations:** 1 AMEE

This review has been migrated. The reviewer awarded 5 stars out of 5 Anyone who works with translators will understand the difference between translation and interpretation, the latter providing a much more holistic approach to the understanding of the translated word. When dealing with sensitive issues, Relationship-centered communication (RCC) skills are vital and training is essential.The authors have modified this paper to a high level and I have no doubt that this paper has been improved and is of great value to those working in the field

Reviewer Expertise:

NA

No decision status is available

# Reviewer report: Reviewer response for version 1

**DOI:** 10.21956/mep.18985.r27209

**Published:** 2018-08-30

**Authors:** Anita Laidlaw

**Affiliations:** 1 University of St Andrews

This review has been migrated. The reviewer awarded 3 stars out of 5 This paper would be of interest to those involved in development of clinical communication training, particularly those considering a training session on the use of medical interpreters. Additionally, it highlights a way to involve stakeholders in the design of training. A workshop for interpreters within a single, academically affiliated, safety net system was designed to elicit discussion around the topic of optimising relationship centred care training for clinical staff. Analysis of the discussion within the workshop was carried out. Response rate was reasonable and the spread of languages represented in attendees at the workshop was broad. Within the introduction it would be helpful to: • Describe how relationship centred care differs from, or overlaps with, person centred care, a term many readers will be more familiar with. • Clarify safety-net care or system. This may be specific to health system structure and needs to be outlined. • Specify whether aims related to communication within consultations involving interpreters. Reported results included points specific to consultations involving interpreters and some more generic points. If the aim is broader, then a justification of why the views of interpreters specifically may be important would be needed.Methods: the authors need to be clear whether data derived from an audio recording of the workshop or notes written during the workshop, this is an important distinction to make. Results: There was some repetition between tables and results section text, with exactly the same quotes appearing in both. It might create more flow within the paper to exclude quotes which appear within the results text from tables. The discussion highlighted important findings and contextualised them. The last take home message suggests they have shown that interpreters should be included in curricular development and institutional implementation. Perhaps more accurately, they have highlighted how interpreters could be helpful to such curriculum development.

Reviewer Expertise:

NA

No decision status is available

# Reviewer report: Reviewer response for version 1

**DOI:** 10.21956/mep.18985.r27208

**Published:** 2018-08-17

**Authors:** Trevor Gibbs

**Affiliations:** 1 AMEE

This review has been migrated. The reviewer awarded 4 stars out of 5 A very useful paper to read for all involved with the development of communication skills in future physicians. The Relationship-centered communication (RCC) skills training described so well certainly adds an extra parameter to the teaching of communication skills. My own personal view of communication is that it must also involve connection- linking the parties concerned and must involve the "whole body" - the sharing, caring, exploring and resolving issues. I think that this paper takes us to those depths on communication skills training and would recommend it to anyone involved with the teaching of communication skills. As the authors state, the sample set is small and from one institution, so I would hope that the authors cannot expand this interesting piece of researh to a more diverse and larger audience.

Reviewer Expertise:

NA

No decision status is available
